# Climate change consequences on the systemic heart of female *Octopus maya*: oxidative phosphorylation assessment and the antioxidant system

**DOI:** 10.1242/bio.060103

**Published:** 2024-05-16

**Authors:** Ana Karen Meza-Buendia, Omar Emiliano Aparicio-Trejo, Fernando Díaz, José Pedraza-Chaverri, Carolina Álvarez-Delgado, Carlos Rosas

**Affiliations:** ^1^ Departamento de Biotecnología Marina, Centro de Investigación Científica y de Educación Superior de Ensenada, 22860 Ensenada, Baja California, México; ^2^ Departamento de Fisiopatología Cardio-Renal, Instituto Nacional de Cardiología “Ignacio Chávez”, 14080 Ciudad de México, México; ^3^ Laboratorio F-315, Departamento de Biología, Facultad de Química, Universidad Nacional Autónoma de México, 04510, Ciudad de México, México; ^4^ Departamento de Innovación Biomédica, Centro de Investigación Científica y de Educación Superior de Ensenada, 22860 Ensenada, Baja California, México; ^5^ Laboratorio de Ecofisiología Aplicada, Unidad Multidisciplinaria de Docencia e Investigación, de Sisal, Facultad de Ciencias, Universidad Nacional Autónoma de México, 97356 Puerto de Abrigo, Sisal, Yucatán, México

**Keywords:** Adult *Octopus maya*, Cephalopods, Mitochondria, Thermal stress, ATP, Respiratory control

## Abstract

There is evidence that indicates that temperature modulates the reproduction of the tropical species *Octopus maya*, through the over- or under-expression of many genes in the brain. If the oxygen supply to the brain depends on the circulatory system, how temperature affects different tissues will begin in the heart, responsible for pumping the oxygen to tissues. The present study examines the impact of heat stress on the mitochondrial function of the systemic heart of adult *O. maya*. The mitochondrial metabolism and antioxidant defense system were measured in the systemic heart tissue of female organisms acclimated to different temperatures (24, 26, and 30°C). The results show that acclimation temperature affects respiratory State 3 and State 4o (oligomycin-induced) with higher values observed in females acclimated at 26°C. The antioxidant defense system is also affected by acclimation temperature with significant differences observed in superoxide dismutase, glutathione S-transferase activities, and glutathione levels. The results suggest that high temperatures (30°C) could exert physical limitations on the circulatory system through the heart pumping, affecting nutrient and oxygen transport to other tissues, including the brain, which exerts control over the reproductive system. The role of the cardiovascular system in supporting aerobic metabolism in octopus females is discussed.

## Teaser

**Summary:** This study addresses the impact of rising temperatures on the cardiac mitochondria of *Octopus maya*, explaining for the first time how temperature probably controls the reproduction of this octopus species.

## INTRODUCTION

Currently, climate change is influencing ocean temperatures, causing an effect on organisms during all life stages with changes in abundance and their distribution patterns, which has resulted in variations in species traits and the establishment of functional diversity (Sunday et al., 2011, 2019). Because the bodily functions of organisms operate within a specific thermal range, the consequences of environmental temperature on ectotherms are of concern, as their body temperature fluctuates with that of their environment (Dickson and Graham, 2004; Guderley and St-Pierre, 2002). Aspects such as temperature tolerance and its dependent effects on performance are central to the thermal biology of ectotherms (Schulte et al., 2011), and their ability to adapt to such fluctuations depends on biochemical, metabolic, and behavioral processes that are altered to maintain homeostasis (Hochachka and Somero, 1968, 2002).

In marine ecosystems, cephalopods are key components due to their vast capacity to be predators or prey in coastal, pelagic, and deep-sea environments (Guimaro et al., 2021; Krstulovíc and Vrgoc, 2009). Cephalopods also occupy environments with high physical and chemical variability from coastal zones to cold, stable and dark environments in the deep sea (Barratt et al., 2007; Daly and Peck, 2000; Leite et al., 2009). Due to their inherently high energy efficiencies, octopuses present high growth rates because of their ability to sustain continuous hyperplastic and hypertrophic muscle growth (Semmens et al., 2004), exhibiting exponential mass increase over much of their short lifespans (Moltschaniwskyj, 2004). Because growth costs determine metabolism, the octopus requires a high metabolic rate that allows for the production of energy needed to sustain their high growth rates (Tan et al., 2019). As a consequence, the high metabolic rates that cephalopods in general, and octopuses in particular, have are linked with a high production of reactive oxygen species (ROS), which in turn are regulated through antioxidant defense mechanisms that are common in invertebrates (Parisi et al., 2021; Rahaman and Rahaman, 2021; Spreitzenbarth and Jeffs, 2021).

Another physiological characteristic of cephalopods is the relationship between metabolic rate, the respiratory system and the circulatory system, which allows them to maintain high metabolic energy production (Melzner et al., 2007). Although there are large differences among cephalopods, e.g. between octopods and squid (Tan et al., 2019), the metabolism of most cephalopods exceeds the rates found among other invertebrate groups that also rely on hemocyanin for oxygen transport (Pörtner, 1995; Strobel et al., 2012). In cephalopods, the oxygen-carrying capacity of blood is limited by its viscosity. While fish blood binds 4-5 mmoles O_2_ l^−1^, cephalopods bind only 1 to 2 mmoles O_2_ l^−1^, implying they have to pump large amounts of blood to satisfy all the oxygen demands these fast-growing animals require (O'Dor and Wells, 1987). In this context, in warm conditions, an increase in oxygen demand is expected, with the consequent increase in pumping blood and increase in the work of hearts directed to satisfy oxygen demands in tissues. When the temperature increases the availability of oxygen for mitochondrial metabolic processes decreases (Abele et al., 2007), and the gene expression of molecules linked to the regulation of harmful ROS is insufficient, with the consequent oxidative stress (Oliveira and Schoffen, 2010; Vinagre et al., 2014).

ROS are probably the most damaging molecules and are produced by incomplete oxygen reduction (Chua et al., 2010). ROS is a collective name that encompasses all oxygen-derived oxidant species (Constantini et al., 2010), such as the radical superoxide (O_2_^•−^), hydrogen peroxide (H_2_O_2_) and the hydroxyl radical (OH^•^); highly reactive molecules, which oxidize biomolecules without specificity giving rise to deleterious cellular processes, such as loss of fluidity of the mitochondrial and plasma membranes, anormal gene expression, reduction in enzyme activity and the arrest of anabolic processes (Passos and Zglinicki, 2006). Also, ROS affects the functioning of the mitochondrial extracellular matrix, thus compromising systemic functioning, which is why the cumulative deleterious action of free radicals has been linked to cellular damage of invertebrates (Feidantsis et al., 2021). Mitochondria can metabolize almost all of the oxygen entering cells; however, about 5% remain as free radicals causing oxidative stress (OS). OS is generated by the leakage of partially reduced, highly reactive oxygen atoms during the transport of electrons through enzyme complexes (mainly I and III) of the respiratory chain in the inner mitochondrial membrane (Chandel et al., 1998; Efremov et al., 2010), and is the result of the imbalance in the prooxidant-antioxidant homeostasis, where oxidation exceeds the antioxidant system (Johnstone et al., 2019; Madeira et al., 2012, 2016a,b).

In invertebrates, high temperatures induce overproduction of ROS and reactive nitrogen species (RNS), disrupting cell molecules through oxidation and nitration, respectively (Abele et al., 2007; Baag et al., 2021; Repolho et al., 2014; Rodríguez-Fuentes et al., 2017). To neutralize ROS, the antioxidant defense system operates at three levels of action (Storey, 1996); (1) prevention of its formation, (2) systemic elimination, and (3) repair of affected cellular components. In antioxidant defense mechanisms, the O_2_^•−^ anion is reduced to H_2_O_2_ spontaneously or by the action of the enzyme superoxide dismutase (SOD); which can be converted to OH^•^ via the Haber-Weis reaction (O_2_^•−^+H_2_O_2_→ O_2_+OH^−^+OH^•^) a transition pathway catalyzed by the presence of Fe^+2^ or Cu^+2^ (therefore, the segregation or chelation of these elements represents a key factor to prevent oxidative stress; Balla et al., 1992) in the Fenton reaction (Fe^2+^+H_2_O_2_→Fe^3+^+OH^−^+OH^•^; Halliwell, 2012).

The H_2_O_2_ produced in this way can also be decomposed into O_2_ and H_2_O by the heme-dependent enzyme catalase (CAT), or used to oxidize substrates (e.g. amino acids, pathogens; Storey, 1996) with the selenium-dependent enzyme glutathione peroxidase (GPx), or with the enzyme peroxiredoxin (Prx) (Di Giulio et al., 1989). Glutathione is used as a substrate for antioxidant enzymes, and it can eliminate O_2_^•−^ and OH^•^. It is also important for vitamin E regeneration and reactivation of inhibited proteins for oxidation (Halliwell and Gutteridge, 2008). In cephalopods an imbalance in the prooxidant-antioxidant homeostasis has been detected in warming conditions even in embryos (Domínguez-Castanedo et al., 2023; Kuan et al., 2022; Repolho et al., 2014), juveniles (Vargas-Abúndez et al., 2023) and adults (Meza-Buendía et al., 2021).

The first mechanism that restricts thermal tolerance in cephalopods is oxygen transport as a mismatch exists between oxygen demand and the ability to supply it to the tissues in high temperatures. (Melzner et al., 2007; Pörtner, 2001; Pörtner and Knust, 2007; Pörtner et al., 2017). In addition, it has been observed that the extent of thermal tolerance arises from limitations imposed by mitochondrial function and its density (Pörtner, 2002). Then, mitochondrial dysfunction (via damage provoked unbalanced between ROS and antioxidant defense mechanisms) may trigger the energy balance disruption, which in turn affects cellular process related to growth and reproduction (Jutfelt et al., 2024; Lemieux and Blier, 2022; Meza-Buendía et al., 2021).

Although the mechanisms underlying cardiac failure have not been determined yet for many species of aquatic ectotherms, a lower mitochondrial ATP supply – essential for cardiac function – has been suggested as one of the mechanisms that explains their thermal limits (Ekström et al., 2023; Pörtner and Knust, 2007). For that reason, cardiac mitochondria, and their ability to produce energy, have been used to evaluate the thermal tolerance in some fish, bivalves, and cephalopod species (Ferreira et al., 2014; Hraoui et al., 2021; Iftikar and Hickey, 2013; Muir et al., 2022; Oellermann et al., 2012; Rodríguez-Zavala and Moreno-Sánchez, 1998).

*Octopus maya* is a tropical species that inhabits the continental shelf of the Yucatan Peninsula located at the entrance of the Gulf of Mexico (Voss and Solis-Ramirez, 1966). Their optimal temperature range is from 22 to 26°C (Angeles-Gonzalez et al., 2017), while at 30°C a reduction of energy destined for reproduction has been determined (Juárez et al., 2015, 2016; Meza-Buendía et al., 2021). To our knowledge, until now no reports were available indicating the thermal sensitivity of the cardiovascular system more accurately in a tropical cephalopod species. In *Sepia officinalis*, it was observed that the heart mitochondria of this temperate species displayed a notable resistance to temperature changes across a broad thermal range. This resilience partially accounts for the species’ wide thermal tolerance (Oellermann et al., 2012). For its part, we have been observing that *O. maya* (Voss and Solís-Ramírez, 1966) is particularly sensitive to high temperatures, which suggests that its cardiovascular system could be less tolerant than *S. officinalis*. It explains, at least in part, the relatively limited thermal tolerance observed in embryos (Caamal-Monsreal et al., 2016; Domínguez-Castanedo et al., 2023), juveniles (Noyola et al., 2013a,b; Vargas-Abúndez et al., 2023) and adults (Domínguez-Estrada et al., 2022; Juárez et al., 2015, 2016, 2022; Meza-Buendía et al., 2021; Pascual et al., 2019).

During the reproduction of *O. maya* and *Illex argentinus* it has been observed that many metabolic pathways work to improve nutritional condition, producing an increase in the feeding rate, and accumulating reserves that are later used during parental care of eggs in the case of females, and in the continuous production of sperm and spermatophores in males (Caamal-Monsreal et al., 2015; Lin et al., 2019; Tercero et al., 2015). Furthermore, neuronal mechanisms that regulate glucocorticoid metabolism are operating to maintain a high metabolic rate (Juárez et al., 2019). For their part, Meza-Buendía et al. (2021) demonstrated that at high temperatures (30°C), the physiological mechanisms involved in energy production in *O. maya* adults were not able to satisfy the additional energy demanded for reproduction at this temperature. When studies were carried out on the effects of temperature on the gene expression of *O. maya* females, it was discovered that in the white body located in the brain of females, high temperatures induce the underexpression of genes from the FMRF-amides family, reducing nutrient transport, proteolysis and in general the response to temperature stimuli, generally affecting the reproduction of this species of octopus. But how does temperature affect the expression of genes in the brains of adult females? If the brain, like all tissues, requires oxygen to meet energy demands through the production of ATP, and the circulatory system is primarily responsible for transporting oxygen to the tissues, it is possible to assume that the systemic heart of an octopus, through the thermal limits of mitochondria, could be limiting the flow of oxygen to tissues, including the brain, causing a cascade of adverse events in the animals. In a first attempt to test this hypothesis the present study was designed to test (1) the effects of thermal acclimation of *O. maya* females on the mitochondrial respiratory states of isolated mitochondria from their systemic heart and (2) the hypothesis indicating that *O. maya* cardiovascular system could be the responsible of their thermal sensitivity.

## RESULTS

### Respiratory rate in State 3

**Fig. 1.**
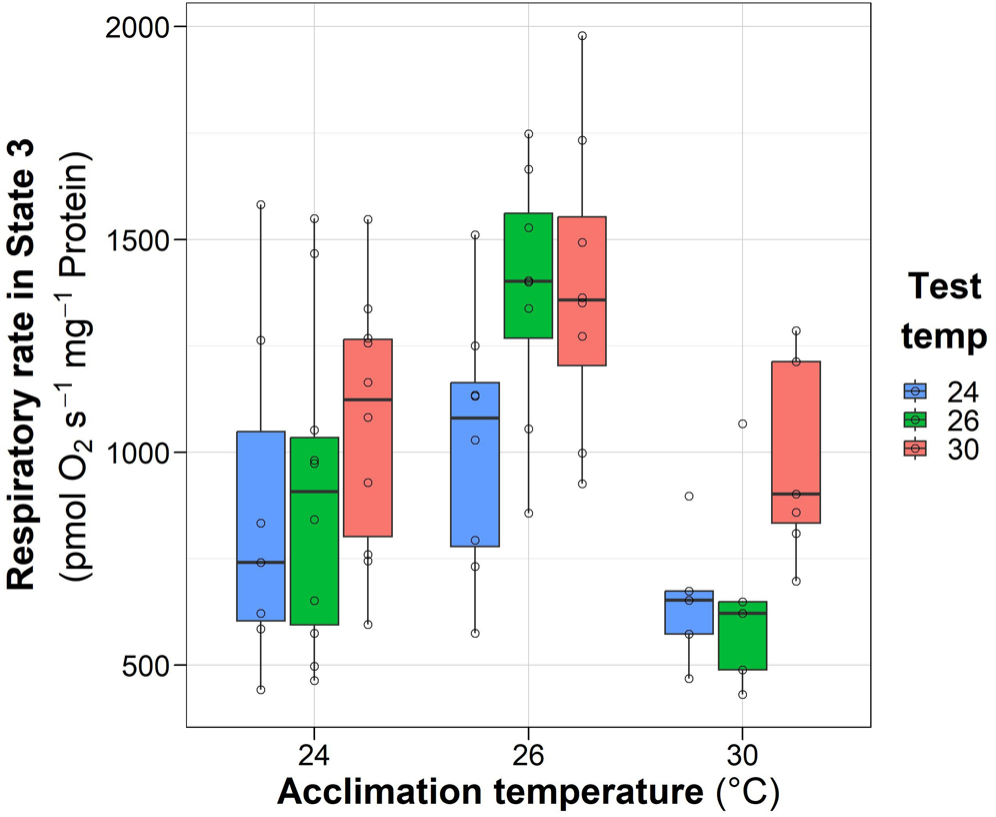
**Effect of acclimation temperature of *O. maya* females on the respiratory rate obtained in State 3 of cardiac mitochondria exposed at 24°C (*n*=7, 10 and 10), 26°C (*N*=8, 8, 8,) and 30°C (*N*=5, 5, 7) (test temperature).** Box and whiskers show the median, 25 and 75% interquartile limits, and minimum and maximum values, respectively. Individual data points are shown as open circles. State 3: referred to oxygen consumption in the presence of exogenous substrates (proline) and adenosine triphosphate (ADP) and obtained after the ROX (residual mitochondrial respiration) correction.

Acclimation temperature had a significant effect (*P*<0.001) on the respiratory rate in State 3, with the highest values obtained in the acclimation group 26°C compared with the 24°C and 30°C groups, according to post hoc Tukey test (*P*<0.05) (Fig. 1, Table 1). Tested temperatures had also significant differences on State 3 respiration, with the highest values obtained when samples were tested at 30°C compared to 24°C (Fig. 1, Table 1). No interaction effects between acclimation and test temperatures were observed (*P*>0.05).

**Table 1.**
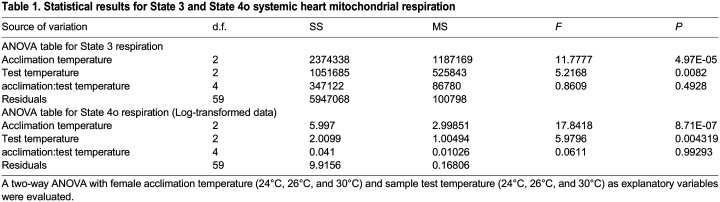
Statistical results for State 3 and State 4o systemic heart mitochondrial respiration

### Respiratory rate in State 4o

**Fig. 2.**
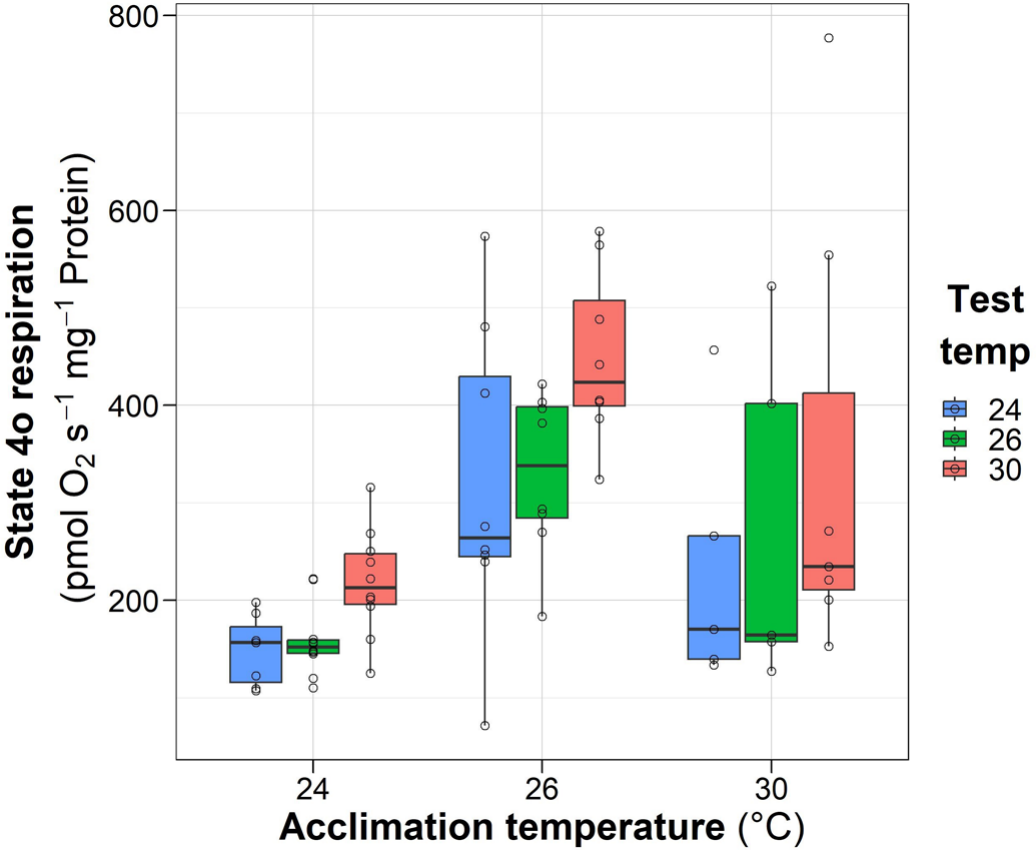
**Effect of acclimation temperature of *O. maya* females on the respiratory rate obtained in State 4o of cardiac mitochondria exposed at 24°C (*n*=7, 10 and 10), 26°C (*N*=8, 8, 8,) and 30°C (*N*=5, 5, 7) (test temperature).** Box and whiskers show the median, 25 and 75% interquartile limits, and minimum and maximum values, respectively. Individual data points are shown as open circles. State 4o – oligomycin induced – refers to oxygen consumption after adenosine triphosphate (ATP) synthase inhibition, reflecting the electron transport chain activity. State 4o was corrected for the respiratory state ROX (residual non-mitochondrial respiration).

A similar result was obtained for respiratory rate in State 4o as for State 3, namely that both acclimation and test temperatures had statistically significant differences (*P*<0.05) without any interaction effect (*P*>0.05) (Fig. 2, Table 1). Likewise, samples from females acclimated to 26°C showed the highest respiratory values, followed by the 30°C and 24°C groups. Tested temperatures showed the highest values as well when samples were tested at 30°C, compared to both 26°C and 24°C (Fig. 2, Table 1).

### OXPHOS capacity

**Fig. 3.**
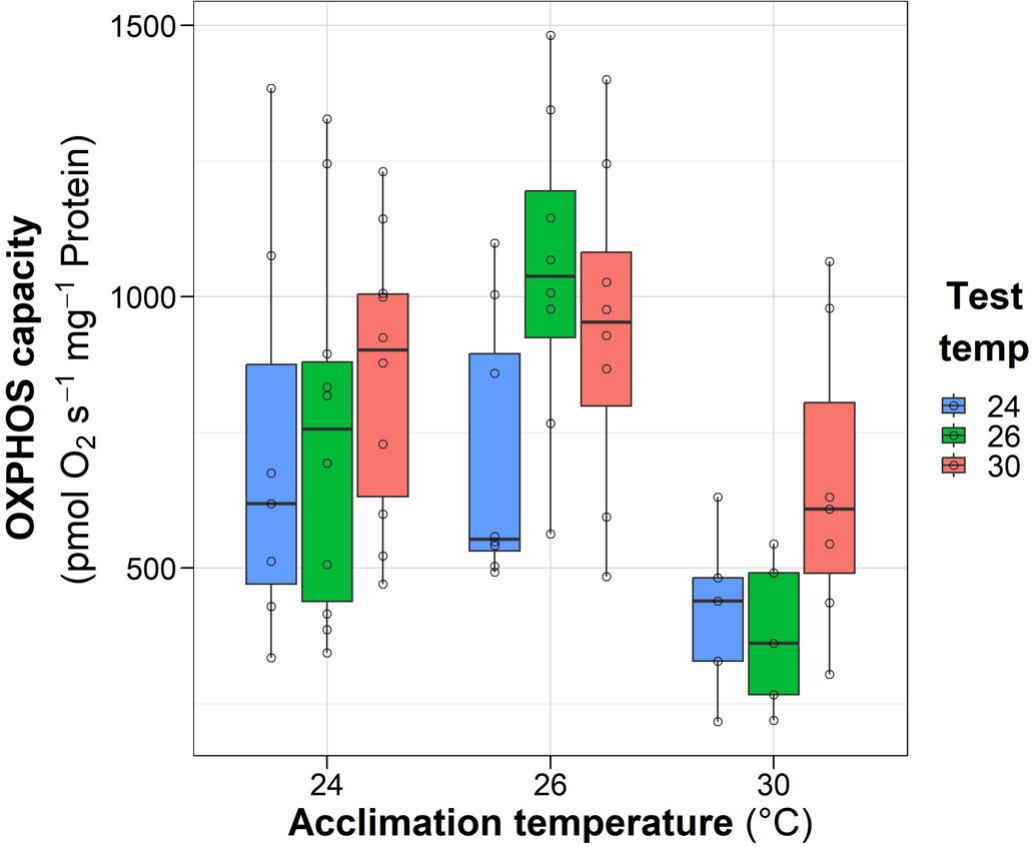
**Effect of acclimation temperature of *O. maya* females on the respiratory rate obtained in the OXPHOS capacity of cardiac mitochondria exposed at 24°C (*n*=7, 10 and 10), 26°C (*N*=8, 8, 8,) and 30°C (*N*=5, 5, 7) (test temperature).** Box and whiskers show the median, 25 and 75% interquartile limits, and minimum and maximum values, respectively. Individual data points are shown as open circles.

Female acclimation temperature caused a significant reduction in ATP production with the lowest values in the mitochondria of females acclimated at 30°C compared to those obtained from females acclimated at 24°C and 26°C (Fig. 3; *F*=9.4; *P*=0.0002; *n*=68). No significant difference (*P*>0.05) was recorded between the test temperatures of each one of the thermal acclimations.

### Respiratory control ratio

**Fig. 4.**
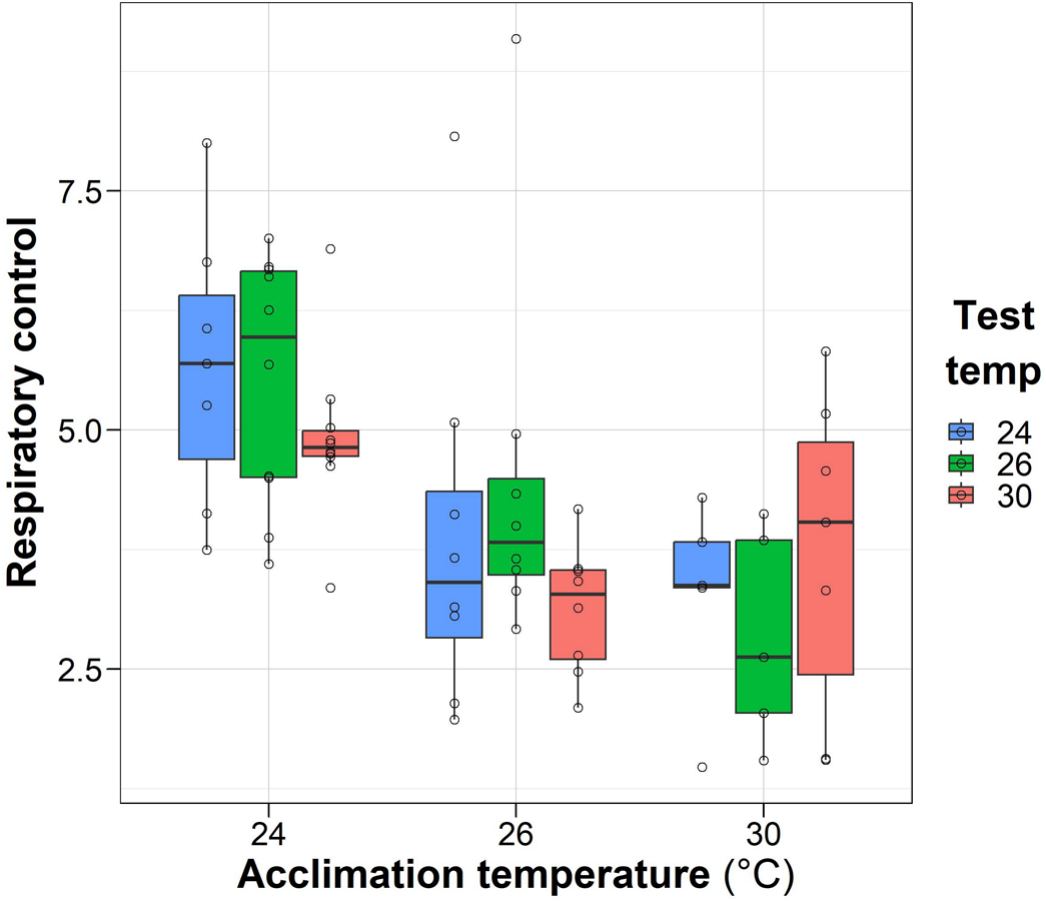
**Effect of acclimation temperature of *O. maya* females on respiratory control of cardiac mitochondria exposed at 24°C (*n*=7, 10 and 10), 26°C (*N*=8, 8, 8,) and 30°C (*N*=5, 5, 7) (test temperature).** Box and whiskers show the median, 25 and 75% interquartile limits, and minimum and maximum values, respectively. Individual data points are shown as open circles.

The respiratory control ratio (RCR) decreased exponentially at higher acclimation temperatures (Fig. 4). The two-way ANOVA indicated a significant decrease (*F*=12.6, *P*<0.001) in respiratory control associated with higher temperatures. Tested temperature of samples did not significantly (*F*=0.8, *P*>0.05) affect the RCR, and no interaction effect was observed (*F*=1.14, *P*>0.05).

### Antioxidant system, oxidative damage, and AChE and CbE activity

**Fig. 5.**
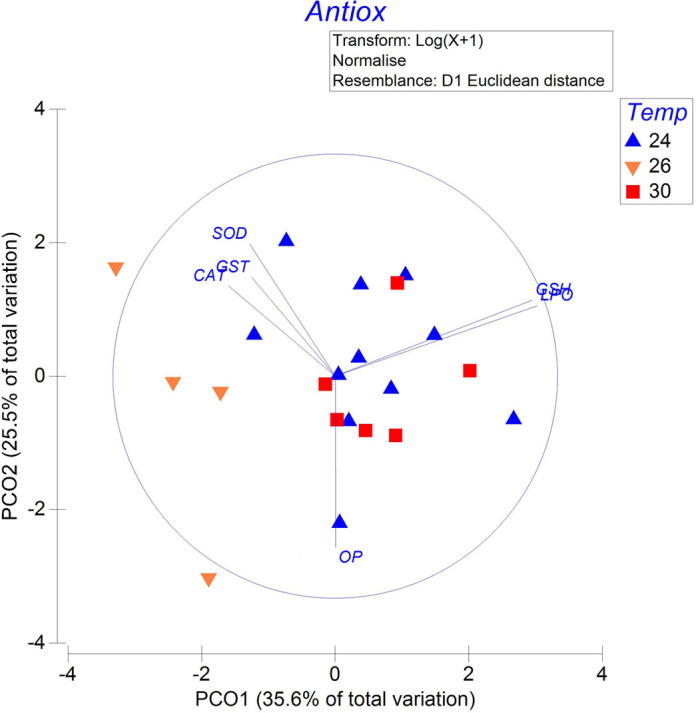
**Principal coordinate analysis (PCoA) of acclimation temperature (24°C, *N*=11; 26°C, *N*=4; and 30°C, *N*=6).** Effect on the antioxidant system (SOD, superoxide dismutase; CAT, catalase; GST, glutathione S-transferase; GSH, total glutathione) and oxidative damage (LPO, lipoperoxidation; OP, oxidized proteins) in the systemic heart of *O. maya* females.

The antioxidant system measured in the systemic heart of *O. maya* females was affected by acclimation temperature (Fig. 5; Table S1). The PCO showed higher PO levels in the hearts of females acclimated at 30°C. In contrast, samples from females acclimated at 24°C and 26°C showed that in those hearts OD generally remained with higher activity than that recorded in hearts from females acclimated at 30°C (Fig. 5; SS=38.08; MS=19.045; Pseudo-*F*=4.1851; *P*=0.001).

**Fig. 6.**
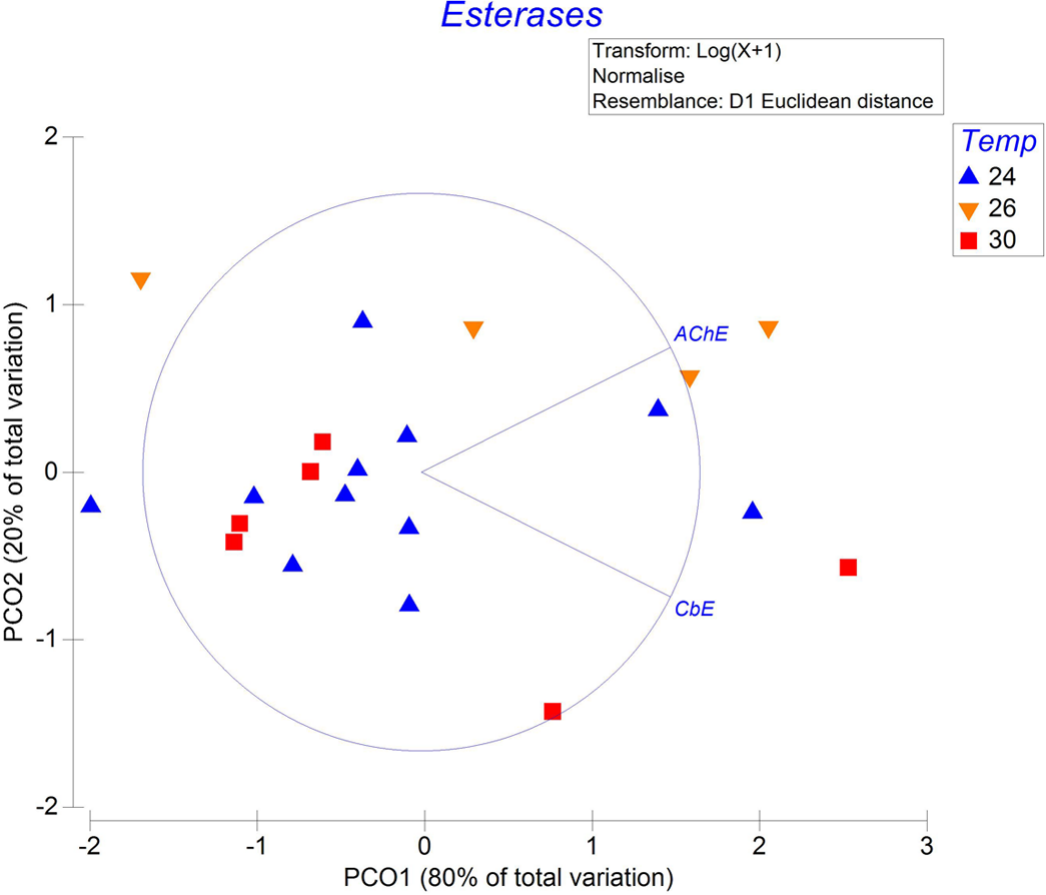
Principal coordinate analysis (PCoA) of acclimation temperature (24°C, *N*=11; 26°C, *N*=4; and 30°C, *N*=6) effect on esterases (AChE: acetylcholinesterase; CbE: carboxylesterase) of the systemic heart of *O. maya* female.

No temperature effect was recorded on esterase levels in the systematic hearts of *O. maya* females (Fig. 6; SS=5.74; MS=2.87; Pseudo-*F*=1.51; *P*=0.001, Table S2).

## DISCUSSION

Previous studies demonstrated that the circulatory system – dependent on the systemic heart of adult *O. maya* females – could be limited by high temperature (Meza-Buendía et al., 2021). In the present study, the heart of females acclimated to 30°C may be limiting hemolymph pumping due to the reduced mitochondrial activity, in turn limiting oxygen transport to the rest of the tissues and organs. This observation would explain, at least in part, why adult *O. maya* exposed to 30°C do not have sufficient metabolic energy available to sustain reproductive requirements (Meza-Buendía et al., 2021). Decreases in mitochondrial function at high temperatures have been reported to be a critical factor in determining upper thermal limits in ectothermic invertebrates (Blier et al., 2014), conditioning the energy available for growth (Dawson et al., 2022; Salin et al., 2016) and reproduction (Meza-Buendía et al., 2021).

Therefore, the relevant pathways used by different species and tissues should be identified to determine how they respond to short-term (acute effects) and long-term (adaptation) temperature changes. In the present study, exposure to acute temperature changes in the mitochondria of acclimated animals provided a clearer picture of the potential effects of temperature on the immediate responses and adaptive capacity of the cardiovascular system of *O. maya* females*.* As an immediate response, the results showed that the S3 respiratory rate of cardiac mitochondria obtained from females acclimated to 24 and 30°C increase their oxygen consumption exponentially with the increase in the test temperature, otherwise for females acclimated to 26°C. S3 reflects oxygen consumption in an ATP production state in which many enzymes are involved – including those of the tricarboxylic acid cycle – electron transport system (ETS), and ATP synthase activity. Thus, upon acute exposure, all the components involved present an exponential type of increase because of the thermodynamic effects that dominate their molecular kinetics (Aledo et al., 2010; Schulte, 2015). Therefore, the results obtained now indicate that all components involved in S3 have the necessary molecular plasticity to respond exponentially, at least in females acclimated to 24°C and 30°C. Studies performed in the cardiac ventricles of several fish species (*Anarhichas lupus*, Lemieux et al., 2010; *Fundulus heteroclitus macrolepidotus*, Fangue et al., 2009; *Oreochromis mossambicus*, Schnell and Seebacher, 2008; *Notolabrius celidotus*, Iftikar and Hickey*,* 2013; *Oncorhynchus mykiss*, Michaelsen et al., 2021, among others) have reported exponential increases in S3 oxygen consumption with temperature; these increases have indicated that as in *O. maya*, mitochondria have some thermal plasticity, as long as the exposure interval does not exceed the thermal limits of the molecules that compose the heart mitochondrial systems of those species. When mitochondria are exposed to temperatures beyond the thermal plasticity threshold, mitochondrial oxygen consumption of these species declines or is maintained, indicating that cardiac mitochondria are no longer functional, as observed in females acclimated to 26°C (Schulte et al., 2011).

Further studies in which *O. maya* cardiac mitochondria are exposed to temperatures higher than 30°C should be performed to establish the limits of mitochondrial plasticity and, with it, the response capabilities of the organisms to an acute change in temperature. The systemic hearts at 30°C in the present study showed a lower S3 than those recorded at 24 and 26°C. This result suggests that exposure to 30°C reduced the energy production capacity of the systemic heart of *O. maya* females because at that temperature, all biochemical processes involved in energy production decrease. The results obtained in rainbow trout showed that complex I (NADH dehydrogenase), along with mitochondrial respiration in S3, decreased their activity above 20°C, which limits the cardiac function of the species when the temperature suddenly increases (Michaelsen et al., 2021). Although a detailed information on the effects of temperature on the activity of complexes mitochondrial in the systemic heart of *O. maya* is not currently available, the processes of this nature may be inferred to occur in animals exposed to 30°C. These results suggest that *O. maya* females are not well adapted to maintain the cardiac activity of the systemic heart at 30°C, at least for a 30-day period.

During S4o measurements, oxygen flux is maintained mainly to compensate for proton leakage at a high chemiosmotic potential when ATP synthase is inactive, which generally increases with temperature (Gnaiger, 2009). The results showed that S4o exposure to acute temperature changes in any of the acclimations females (24°C, 26°C and 30°C) causes an increase that follows the shape of an exponential curve following, as mentioned previously, the molecular dynamics imposed by temperature. Considering that the highest S4o respiration values were recorded during acclimation to 30°C, it could be inferred that the ETS of the systemic hearts of *O. maya* is not well adapted to function, at least up to 30°C. Moreover, the results obtained now concerning the presence of ROS suggest that under such thermal conditions, an increased proton leakage affected ATP production (Salin et al., 2016; Schulte, 2015).

Studies performed on isolated mitochondria from fish hearts, red muscle, liver (Fangue et al., 2009; Hardewig et al., 1999; Hilton et al., 2010) and gills of Antarctic bivalves (Pörtner et al., 1999) have shown that proton leakage is linked to increased mitochondrial membrane fluidity because of heat stress and reduced efficiency in energy production. Moreover, Iftikar and Hickey (2013) found that ATP production in cardiac fibers of *Notolabrus celidotus* measured by fluorometry is limited at 25°C and accompanied by an increase in inner membrane proton leakage and altered flux rates in the ETS. Evidently, at high suboptimal temperatures, the integrity of mitochondrial membranes is lost and consequently the capacity for ATP synthesis.

Although the rate of ROS production in cardiac mitochondria was not measured in the present study, the antioxidant activity and oxidative damage (OD) indicator molecules from homogenized cardiac tissue were evaluated. From these results, it is interesting to note that a more significant increase in oxidized proteins (OP) levels was observed in the cardiac tissue from *O. maya* females acclimated to 30°C, which indicated that ROS formation was stimulated in this thermal environment. Similar results have been reported in mitochondria isolated from the mantle of *Mya arenaria*, where heat stress caused an increase in ROS production (Abele et al., 2002), suggesting a close relationship between mitochondrial dysfunction and oxidative damage associated with proton leakage, which in *M. arenaria* and *O. maya* is stimulated by heat stress.

Interestingly, the results obtained in the present study suggest that the nervous system involved in systemic heart activity was not altered by temperature. AChE is associated with the breakdown of acetylcholine responsible for terminating postsynaptic signal transmission. Acetylcholine is a neurotransmitter released by parasympathetic nerves that regulate changes in heart rate and the necessary contractility for proper cardiovascular function (Roy et al., 2013). Although it is still not clear how cardiac function is regulated by the nervous system in *O. maya*, the results obtained now suggest that the heart rate must have been adequately synchronized – at least for the time the females were in acclimation (30 days) – despite the reduction in efficiency of mitochondrial energy production (respiratory control) in females exposed to 30°C.

It is interesting to note that *O. maya* mitochondria have a higher thermal sensitivity than observed in other cephalopod species. The aerobic capacity (State 3) of heart mitochondria from *Sepia officinalis* was not affected by thermal acclimation measured over a range from 11 to 21°C (Oellermann et al., 2012). This result suggests that *S. officinalis* heart mitochondria are less thermally sensitive than that from *O. maya* females. Studies performed on fish and crustaceans from tropical and temperate ecosystems (Vinagre et al., 2016) showed that tropical species have a lower acclimation capacity than temperate ones. This result demonstrates that such thermal differences are due to tropical species being closer to their thermal limits than temperate ones. Although the methods evaluating the effects of temperature on *S. officinalis* and *O. maya* heart mitochondrial activity were different, the differences observed in thermal heart sensitivity of cephalopods may be explained by lower acclimation capacity of tropical species (*O. maya*) when compared with temperate species (*S. officinalis*). Thus, in temperate species, a broader acclimation capacity could be linked with the ability of their heart to maintain a high-temperature activity, guaranteeing the oxygen supply to tissues via heart rate (Oellermann et al., 2012).

On the contrary, in tropical species as *O. maya*, the thermal limits appear to be linked with the limited capacity of the heart mitochondria, which cannot maintain a high ATP production in high temperatures with probable consequences in heart rate and oxygenated hemocyanin transport to tissues. Juárez et al. (2015) found that 27°C could be the thermal limit for reproduction in *O. maya* females because at higher temperatures spawning was inhibited. Moreover, those authors observed that temperatures higher than 27°C compromised embryo development.

Despite the additional effects that provoke high temperature on the ovary of *O. maya* females are still not known, possibly, a heart energetically limited by the high temperature may also produce limitations in pumping oxygenated hemolymph, consequently, in oxygen transport to tissues to satisfy the energetic demands that occur in the ovary and oviductal gland – two critical organs involved in the octopus reproduction (Domínguez-Estrada et al., 2022; Juárez et al., 2022; Olivares et al., 2017). In this sense, a question arises: How would the systemic heart mitochondria control reproduction in octopus? A possible answer could be that systemic heart mitochondria are linked with the energy used by the heart to pump oxygenate hemocyanin to the ovary of females.

**Fig. 7.**
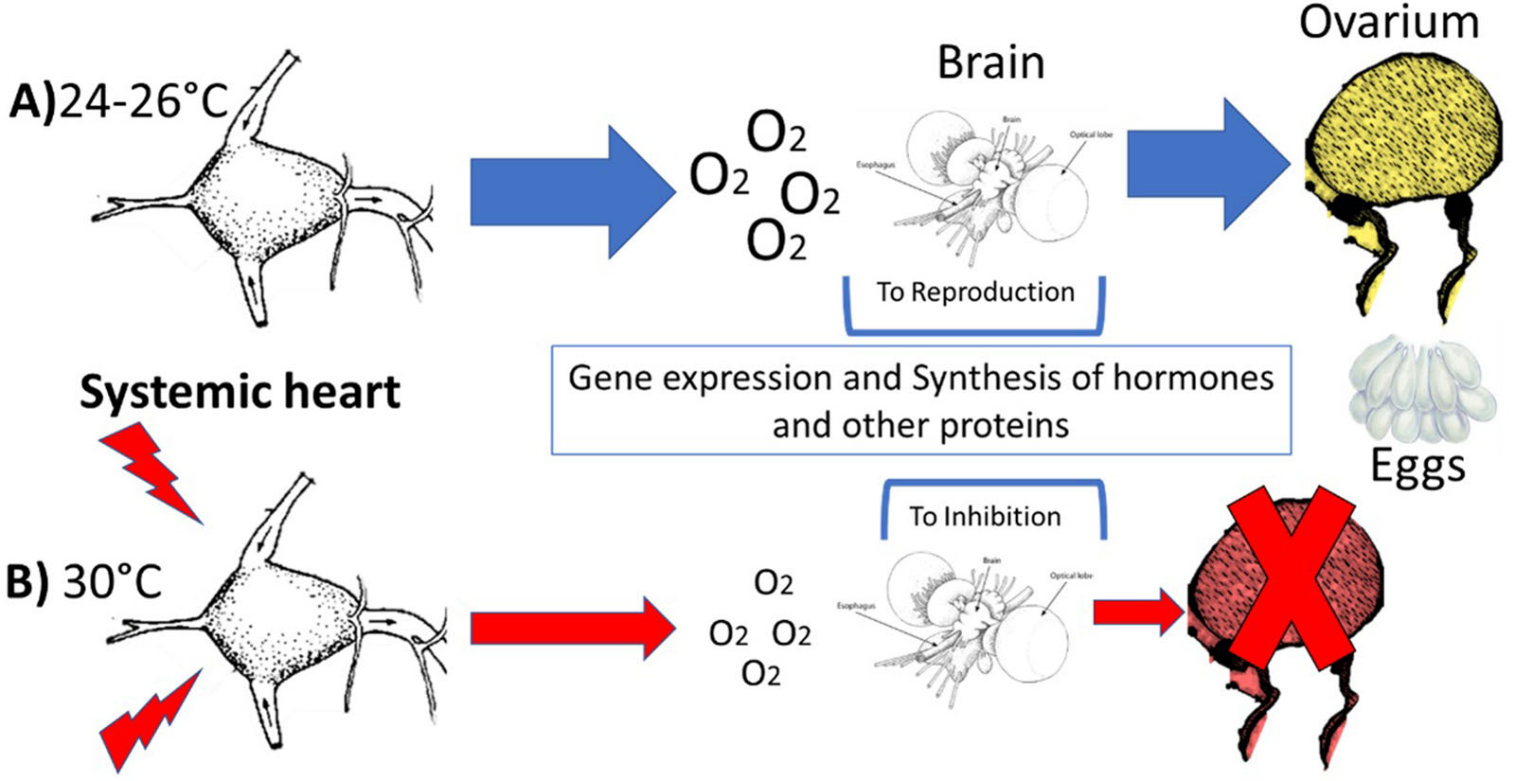
**Hemolymph oxygen is a signal in the brain of octopus females for reproductive regulation.** (A) In optimal temperatures (24-26°C) the systemic heart works optimally, allowing a high oxygen flow to tissues. In the brain, gene regulation stimulates oviducal glands and ovarium to spawn. (B) In high temperatures, the low ATP mitochondrial production affects the heart rate and oxygen flow to tissues. In the brain, the oxygen demand is not satisfied at high temperatures (30°C), provoking gene regulation and the inhibiting of the reproductive process in the ovarium and the oviducal gland.

Consequently, less oxygen in hemolymph implies less energy in the ovary and changes in yolk composition, the number of eggs, and fewer embryos, probably with fewer reserves and smaller than observed in embryos from non-thermally stressed females. In such cases, thermally stressed females spawn, even when embryos are at thermal risk. However, the results obtained in the laboratory have demonstrated that in temperatures higher than 27°C, the octopus spawn is inhibited, suggesting that other more complex processes regulate *O. maya* reproduction during thermal stress (Di Cosmo and Polese, 2014; Domínguez-Estrada et al., 2022; Juárez et al., 2015, 2019; Ventura-López et al., 2022). Considering the previous results, a second question arises: Could the strength of the oxygen flow pumped from the systemic heart be the signal to the brain that triggers the complex regulatory mechanisms directed to preserve the embryos’ integrity, inhibiting spawning at high temperatures? (Fig. 7).

In the last few years, studies have demonstrated that thermal stress induces up-and-down gene regulation directed to inhibit spawning, probably as an adaptation to avoid embryo stress. Although until now, the energy demand involved in gene regulation for reproduction into the brain is not known, our hypothesis is that when the temperature is optimal, the hemolymph oxygen concentration signals the brain to indicate that enough energy is available for the reproductive process (Fig. 7). The opposite signal could be operating in thermally stressed females whose hearts have reached their thermal limits and their capacity to pump oxygenated hemolymph to tissues. At neuronal level, complex mechanisms operate through the regulation of glucocorticoid metabolism, which, added to other proteins of the FMRF-amide family (FMRF; PRQF among others), they stimulate *O. maya* females during the growth phase to maintain a high metabolic rate, ensuring at the same time the ingestion of enough food quantity (Juárez et al., 2019; 2022; Tercero et al., 2015). In optimal thermal conditions genes are downregulated allowing females to regulate their metabolism channeling the available energy to the end of maturation processes and spawn. Under thermal stress, the FMRF-amide family is upregulated, which is one of the responsible proteins for inhibiting spawning (Juárez et al., 2019).

Juárez et al. (2022) also reported in thermally stressed females' overexpression of MYOM and APGW-amide genes. These neuropeptides potentiate the muscular contraction of the oviduct limiting the passage of eggs from the ovary to the oviducal gland, reducing or inhibiting the fertilization rate. Furthermore, the genes LGALS3 and VWC2 were downregulated in thermally stressed females, both genes encoding proteins involved in the union of the spermatozoon to the extracellular matrix of the octopus's egg. These results explain why thermally stressed females have few fertilized eggs when spawning (Juárez et al., 2015) and show how the brain, probably without enough oxygen could be making a control over the spawn.

Meza-Buendía et al. (2021) proposed that 26°C is the optimal extreme temperature for *O. maya* adults. In that thermal condition, *O. maya* males, and females showed their maximum aerobic scope, indicating that animals satisfied their metabolic requirements at their maximum capacity. This result explains why at this temperature, reproduction is better than at low or high temperatures (Juárez et al., 2015, 2022). The results obtained in the present study show that at 26°C cardiac mitochondria of this acclimation group (26°C) have the highest State 3 respiration, indicating that this temperature is where the cardiac activity is the highest (maximum activity of the proton transport chain and ATP production) and consequently for the transport of oxygen to the rest of tissues, including the reproductive ones. Juárez et al. (2015, 2022), Domínguez-Estrada et al. (2022), and Ventura-López et al. (2022) studied gene expression in *O. maya* females exposed to different temperatures. These authors found that in the interval from 24 to 26°C many genes are involved in biochemical processes that ensure the sperm maintenance and egg fertilization success, upregulating in *O. maya* brain and oviductal gland, probably due to high oxygen availability. In this sense, our hypothesis is that when temperature is optima, oxygen signals to the brain, indicating the reproductive female tissues that enough energy is ready for the reproductive process. Studies in fish have demonstrated that heart rate correlates closely with optimum temperature for many physiological processes (Fry, 1947, 1971), which suggests that, as in fish in *O. maya*, a high heart performance is correlated with optimal thermal conditions.

## MATERIALS AND METHODS

### Ethical statements

The protocols followed were approved by the Tics and scientific responsibility commission of the Faculty of Sciences at Universidad Nacional Autónoma de México, (permit number: CEARC/Bioética/25102021).

### Origin of animals

Twenty-seven wild adult female *O. maya* were captured off the coast of Sisal of the Yucatan Peninsula (21° 9′ 55′′ *N*, 90° 1′ 50′′′ W) by the local drift-fishing method known as ‘Gareteo’ (Solís-Ramírez, 1967) and crab *Callinectes sapidus* was used as bait. The octopuses fished were sexually mature with a reproductive system (Ávila-Poveda et al., 2016), and we carried out four fishing trips in April, June, October 2021, and May 2022 (Table S3). During the captures, we kept the octopuses in a 5000-l black circular tank with seawater recirculation until arrival at the laboratory facilities.

### Laboratory conditioning

Before the experimental acclimation treatments, specimens were conditioned for 7 days in 6-m diameter outdoor ponds with aerated seawater (26-30°C) at a stocking density of one animal per m^2^. The ponds were covered with black mesh to reduce direct sunlight by 70% and connected to seawater recirculation systems coupled to protein skimmers and 500-μm bag filters. Open PVC tubes of 50 mm diameter were placed as shelters at a 2:1 ratio per animal. During this conditioning period, the octopuses were individually fed twice daily (0900 and 1700 h) with a squid and crab meat-based paste at a proportion of 8% of their body weight (Tercero et al., 2015). Uneaten food and feces were removed daily.

### Experimental acclimation treatments

After the conditioning period, adult *O. maya* females were distributed individually in 80 l tanks grouped in three acclimation temperatures: ten animals (1170±270 g wet weight) to 24°C; eight animals (924±258 g wet weight) to 26°C and nine animals (1322±298 g wet weight) to 30°C (Fig. S1). The octopuses were maintained under these experimental conditions for 30 days and fed following the same feeding protocol as in the conditioning period. This time was considered, considering that in *O. maya* females, the final sexual maturity, when the eggs develop in the ovary, occurs precisely in the last 30 days before spawning (Ávila-Poveda et al., 2015, 2016). Previous results showed that it is precise during this time when the energetic demands are increased due to the yolk synthesis in the ovarium, which is regulated by the brain (Juárez et al., 2016, 2019, 2022). Each group was maintained in separate tanks connected to independent recirculation systems for each acclimation temperature. The seawater inlet temperature of the recirculation system was maintained at 26°C. Thus, to obtain the temperature of 30°C, it was gradually increased by 1°C per day until it reached and maintained with 1800-watt heaters connected to automatic temperature controllers. The temperature of 24°C was controlled with a titanium chiller (Ultra Temp^®^ Pentair), as well as the air conditioning of the experimental room. Water quality parameters were monitored weekly and maintained at levels suitable for *O. maya* culture (salinity 36±1, >5 mg O_2_ l^−1^, pH>8, NH_4_^+^<0.2 mg l^−1^) and a photoperiod of 12 light/12 dark under a light intensity of 30 lux m^−2^.

### Mitochondrial isolation from cardiac tissue

Mitochondria were obtained from the systemic heart of each octopus within the acclimation groups (24°C, 26°C, and 30°C) after 30 days. Therefore, the bioethical guidelines of the Faculty of Sciences of the UNAM (Mexico) were followed, and to prevent distress and minimize pain in the specimens, they were anesthetized with 3% ethanol dissolved in seawater for an average of 12 to 15 min. Once the lack of response evoked by the stimulus was observed in the octopus, we opened the mantle cavity and extracted the complete systemic heart. This entire procedure was carried out in the shortest possible time to avoid affecting the quality of the mitochondria.

A 0.1 g sample of cardiac tissue was collected and immediately stored at −80°C for enzyme activity assay. The rest of the heart was weighed (g wet weight; Table S4) and used for mitochondrial isolation following the protocol proposed by Meza-Buendia et al. (2022), which procedure was performed cold (4°C). Subsequently, the organ was minced into small pieces and homogenized using a Potter-Elvehjem PTFE mortar and glass tube (Sigma-Aldrich P7859-1EA, MO, USA) operated by a drill at 500 rpm with 2 ml of isolation buffer [500 mM sucrose, 300 mM KCl, 2 mM ethylene glycol-bis (β-aminoethyl)-N, N, N′, N′-tetraacetic acid (EGTA), 25 mM 4-(2-hydroxyethyl)- piperazine-1-ethanesulfonic acid (HEPES), 1.5% w/v fatty acid-free bovine serum albumin (FA-free BSA), pH 7.4 at 20°C, 826 mOsM].

The homogenate was centrifuged at 392 ***g*** (4°C) for 5 min in polycarbonate centrifuge tubes; the supernatant was recovered and centrifuged at 7939 ***g*** (4°C) for 15 min. The supernatant was discarded by decanting, and 2 ml of isolation buffer without FA-free BSA was added to re-suspend the pellet formed. Finally, the sample was centrifuged at 7939 ***g*** (4°C) for 15 min; the resulting supernatant was discarded, and the formed pellet (mitochondrial fraction) was re-suspended with 160 μl of isolation buffer without FA-free BSA. Afterward, 10 μl of each mitochondrial isolate sample was used to determine protein concentration by the Bradford (1976) method. The rest of the sample was kept cold (4°C) for evaluation of mitochondrial respiration and used within the first four hours to obtain better functional responses. Mitochondria from each acclimation group were evaluated at three test temperatures: 24°C, 26°C, and 30°C.

### Mitochondrial O_2_ consumption measurements

Mitochondrial oxygen consumption rates were determined using an Oxygraph-2k™ (O2k, Oroboros Instruments, Austria), which consists of an enclosed respirometer with two 2-ml chambers using a polarographic oxygen sensor to detect oxygen (O_2_) flux of ±1 pmol O₂ s^−1^ ml^−1^. Before starting the determination of oxygen consumption, the oxygen sensors were calibrated at each test temperature: 24°C, 26°C, and 30°C. For the oxygen sensor calibrations, MiR05 respiration medium was used, consisting of (in mM): 0.5 EGTA, 3 MgCl_2_, 60 lactobionic acid, 20 Taurine, 10 KH_2_PO_4_, 20 HEPES, 110 D-Sucrose and 1 g l^−1^ FA-free BSA (pH 7).

Once the O2k system reached the steady state of oxygen consumption of the polarographic oxygen sensor, where the consumption rate is constant, isolated mitochondria (0.6-1.0 mg, previously determined) from the systemic heart were added. Immediately after, proline (Pro) was added at a final concentration of 5 mM to avoid depolarization of the mitochondrial membrane potential; then, adenosine diphosphate (ADP, 1.25 mM) was added to induce OXPHOS-associated respiration state (state 3′, S3′). Oligomycin (0.0025 mM) was added to induce the oligomycin-induced LEAK state (state 4′o, S4′o), which corresponds to the oxygen consumption associated with proton conductance, proton slip, and cation cycling. Finally, rotenone (0.0025 mM) plus antimycin A (0.0125 mM) were added to obtain the residual respiration ROX, which corresponds to residual non-mitochondrial respiration (Fig. S2).

### Mitochondrial parameters

The different respiratory rates measured in the respiratory states were used to calculate mitochondrial parameters: respiratory state S3, respiratory state S4o, respiratory control, and OXPHOS capacity. The mitochondrial respiratory states S3 (S3=S3'-ROX) and S4o (S4=S4'o-ROX) were corrected for the respiratory state ROX. After correction, the respiratory control ratio (mitochondrial coupling) was defined as S3/S4o, while the mitochondrial respiratory rate attributed to OXPHOS (OXPHOS capacity) was defined as S3-S4o. All reported values were normalized by total protein content and were expressed in pmol O_2_ s^−1^ mg protein^−1^ except for the respiratory control ratio.

### Antioxidant defense system (ANTIOX), OD, and acetylcholinesterase (AChE) and carboxylesterase (CbE) activities

Each of the frozen cardiac tissue samples obtained from the different acclimation groups was homogenized in cold 50 mM Triz buffer (pH 7.4) to 100 mg ml^−1^ of tissue in a Potter-Elvehjem homogenizer (Sigma-Aldrich P7859-1EA, MO, USA). A portion of the homogenate was centrifuged at 10,000 ***g*** (4°C) for 5 min, and the supernatant was used for the analysis of the enzymes: SOD, CAT, glutathione-S-transferase (GST), AChE and CbE. The remainder of the homogenate was used to assess total glutathione (GSH), lipid peroxidation (LPO), and carbonyl groups in OPs. All assays were performed in triplicate subsamples.

SOD activity, CAT, GSH, and GST were measured to assess the ANTIOX. SOD is an enzyme that alternately catalyzes dismutation (or partitioning) of the superoxide radical into molecular oxygen and hydrogen peroxide. A Sigma-Aldrich assay kit (19160; USA) was used to evaluate SOD, using Dojindo's highly water-soluble tetrazolium salt, WST-1(2-(4-Iodophenyl)-3-(4-nitrophenyl)-5-(2,4-disulfophenyl)-2H tetrazolium, monosodium salt) that produces a water-soluble formazan dye upon reduction with a superoxide anion. The reduction rate with O_2_ is linearly related to the xanthine oxidase (XO) activity and inhibited by SOD. The CAT was measured using the method of Goth (1991) with a modification by Hadwan and Abed (2015). This method is based on the reduction of H_2_O_2_ levels using the UV method.

GSH was measured with a Sigma-Aldrich Glutathione Assay Kit (CS0260) (USA). This kit utilizes an enzymatic recycling method with glutathione reductase (Baker et al., 1990). The GSH sulfhydryl group reacts with Ellman's reagent, producing a yellow compound read at 405 nm. GST was measured with a Sigma-Aldrich GST assay Kit (CS0410). This enzyme catalyzes the conjugation of the glutathione thiol group to compounds containing electrophilic centers. The kit utilizes a 1-chloro-2,4-dinitrobenzene substrate (CDNB), which conjugates with the glutathione thiol group, forming a compound that can be read at 340 nm.

To evaluate the OD caused by ROS, LPO, and carbonyl groups in OPs were measured. Following the manufacturer's instructions, LPO was evaluated using Peroxi Detect Kit (PD1, Sigma-Aldrich, USA). In this assay, peroxide oxidizes Fe^2+^ ions at acidic pH, forming a colored adduct with Xylenol Orange that is measured at 560 nm; OP was estimated using the 2,4-dinitrophenylhydrazine alkaline protocols developed by Mesquita et al. (2014) and reported in nmol mg^−1^ wet weight. For this assay, 200 μl of 2,4 dinitrophenylhydrazine (10 mM in 0.5 M HCL) was incubated with 200 μl of the sample homogenate and 100 μl of NaOH (6 M). Absorbance was read at 450 nm after 10 min of incubation at room temperature against a blank where an equal volume of homogenization buffer substitutes the protein solution.

For this study, two esterases were measured to evaluate physiological conditions, AChE and CbE. AChE activity was measured using the Ellman et al. (1961) method, adapted to a microplate reader (Rodríguez-Fuentes et al., 2008). Each well contained 10 μl of the enzyme supernatant and 180 μl of 5, 5-dithiobis (2 nitrobenzoic acids; DTNB) 0.5 mM in 0.05 M Tris buffer pH 7.4. The reaction started by adding 10 μl of acetylthiocholine iodide (1 mM). The absorbance change rate at 405 nm was measured for 120 s. CbE activity was measured using ρ-nitrophenyl-α-arabinofuranoside (ρNPA) substrate, as indicated by Satoh and Hosokawa (2001), with some modifications. Each assay included 25 μl of the supernatant and 200 μl of ρNPA; the reaction was recorded at 405 nm for 5 min. SOD, AChE, and CbE activities were reported as mg protein in the sample (Bradford, 1976).

### Statistical analysis

All tests and data analyses were performed with the base packages of the R software, version 4.2.2 (R Core Team, 2022), with the R package ggplot2 (Wickham, 2016) for data visualization. Specific responses of State 3 respiratory rates, State 4o respiratory rates, OXPHOS capacity, and respiratory control at different temperatures were evaluated by two-way analysis of variance (ANOVA), with pairwise comparison where appropriate. Acclimation and test temperatures were determined as factors, and each of the mitochondrial parameters was evaluated as a response variable. All data are presented as mean±s.e.m. unless otherwise indicated. Sample size (*n*) is indicated in the figure captions. Normality was verified with visualization of residuals and homogeneity of variances was verified using the Levene's test, and data were transformed when required.

Variations in enzymes (SOD, CAT, and GST) and no-enzymes (GSH) involved in the ANTIOX system, oxidative damage markers (LPO and OP), and AChE and CbE esterases across acclimation temperatures (24, 26, and 30°C) of adult *O. maya* females were evaluated by the Principal Coordinate Analysis (PCoA). PCoA was calculated from a similarity matrix with dissimilarity measures (Euclidean distance) between each pair of samples. The raw data were pretreated with natural logarithm (Log[X+1]) and z-normalization. Afterwards, the statistical significance of data clusters was tested by multiple ANOVA with permutations (PERMANOVA; Anderson, 2001). The underlying model was a one-way ANOVA with acclimation temperature as a fixed factor with three levels: 24°C, 26°C and 30°C. Unrestricted permutations of raw data with 999 unique permutations were used to generate empirical distributions of pseudo-F values under the null hypotheses (Anderson, 2017).

## Supplementary Material

10.1242/biolopen.060103_sup1 Supplementary information
