# Supplementary material for: Climate change consequences on the systemic heart of female Octopus maya: oxidative phosphorylation assessment and the antioxidant system
Source: Biol Open. 2024 May 16;13(5):bio060103. doi: 10.1242/bio.060103 (PMC11155352; doi:10.1242/bio.060103)
Supplement: Supplementary information [file biolopen-13-060103-s1.pdf]

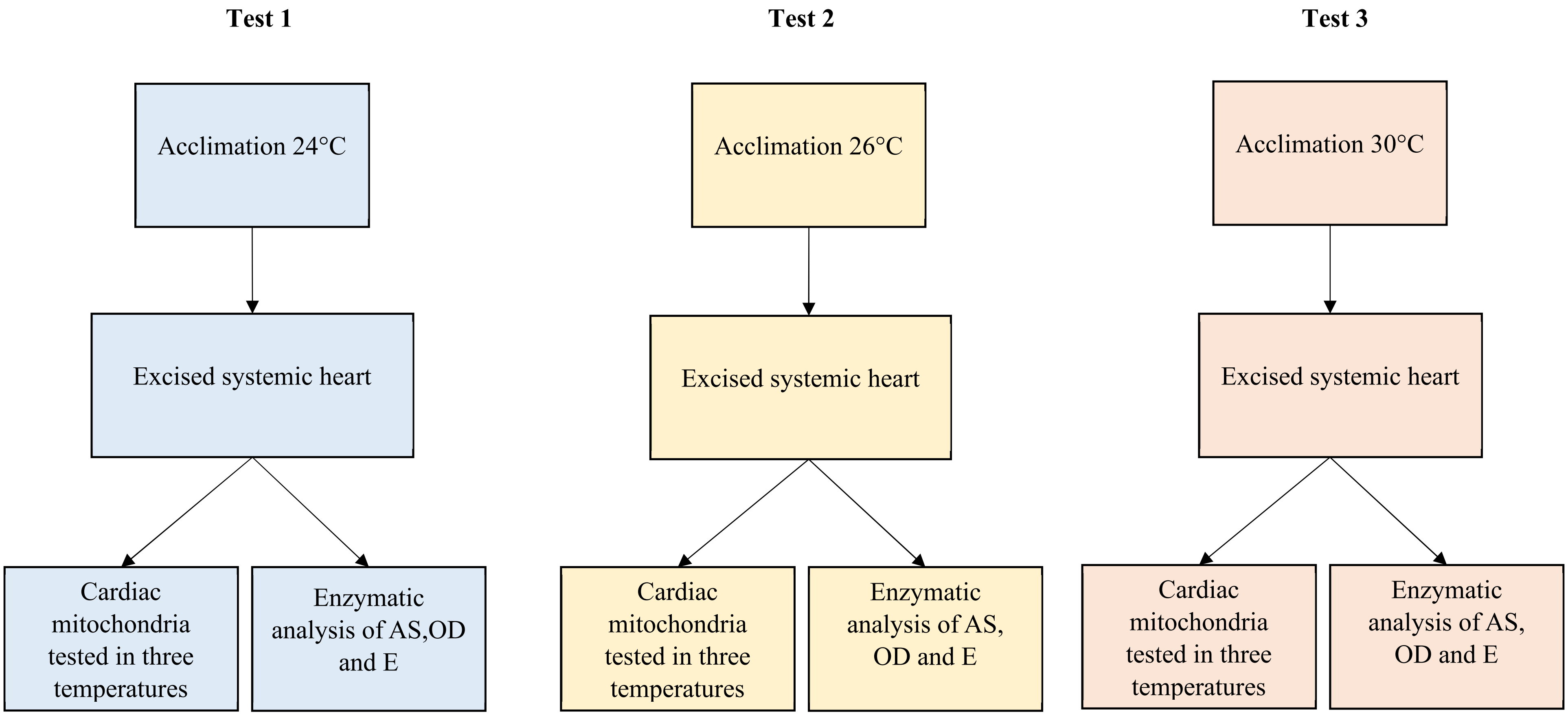

Fig. S1. Schematic of the experimental protocol of the present study. The experimental design is illustrated as a flow chart representing the three experimental runs. AS= antioxioxidant system, OD=oxidative damage, E= esterases.

Biology Open • Supplementary information

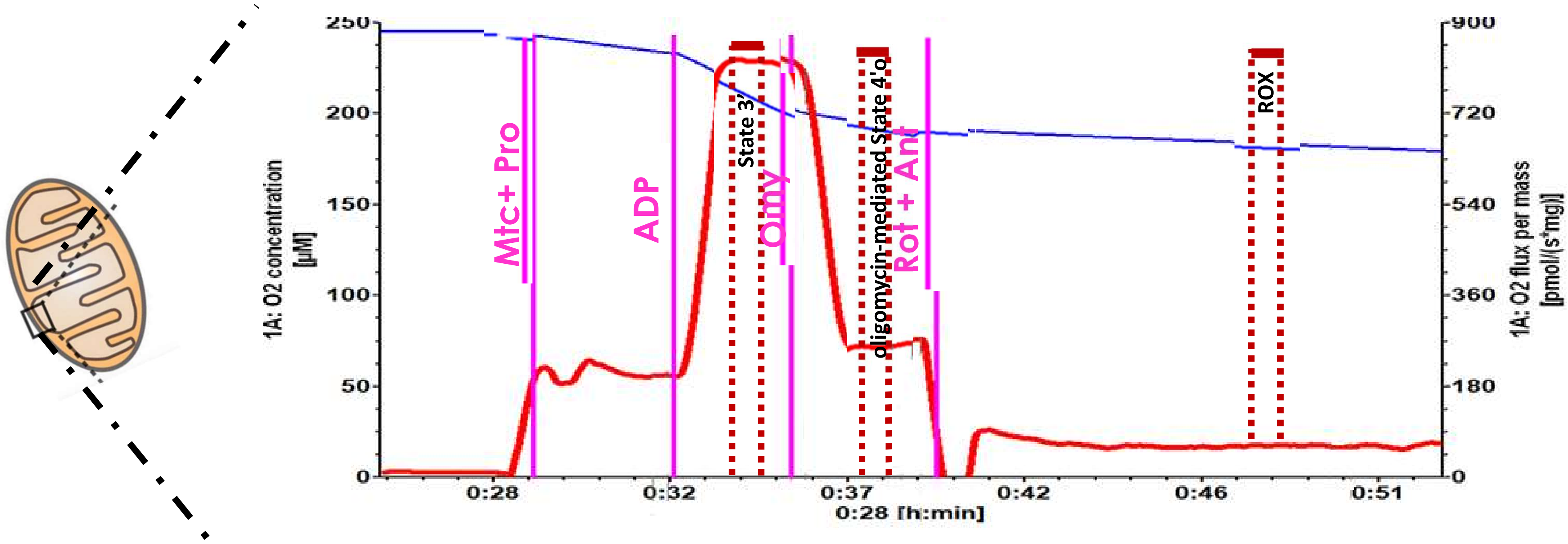

Fig. S2. Schematic representations of the method used to determine the rate of oxygen consumption in each respiratory state (State 3', oligomycin-mediated State 4'o, and ROX); the blue line corresponds to O_2 concentration (µM), while the red line corresponds to oxygen consumption rate normalized by amount of protein (pmol O_2 s^{-1} mg^{-1}). Mtc: mitochondria, Pro: proline, ADP: adenosine diphosphate, Omy: oligomycin, Rot: rotenone, and Ant: antimycin A. ROX= residual non-mitochondrial respiration.

Table S1. PERMANOVA results of the effects of temperature on antioxidant defense mechanisms and oxidant damage of the hearts of *O. maya* adults.

PERMANOVA table of results

Source	df	SS	MS	Pseudo-F	P(perm)	Unique perms
Ac	2	38.089	19.045	4.1851	0.001	999
Res	18	81.911	4.5506			
Total	20	120				

Details of the expected mean squares (EMS) for the model

Source	EMS
Ac	1*V(Res) + 6.381*S(Ac)
Res	1*V(Res)

Construction of Pseudo-F ratio(s) from mean squares

Source	Numerator	Denominator	Num.df	Den.df
Ac	1*Ac	1*Res	2	18

Estimates of components of variation

Source	Estimate	Sq.root
S(Ac)	2.2715	1.5071
V(Res)	4.5506	2.1332

PAIR-WISE TESTS

Term 'Ac'

Groups	t	P(perm)	Unique perms
24, 26	2.3322	0.001	708
24, 30	1.594	0.034	959
26, 30	2.0672	0.005	209

Denominators

Groups	Denominator	Den.df
24, 26	1*Res	13
24, 30	1*Res	15
26, 30	1*Res	8

Average Distance between/within groups

	24	26	30
24	2.6533		
26	4.2772	4.1324	
30	2.8369	4.2868	2.4977

Table S2. PERMANOVA results of the effects of temperature on Carboxyl esterase (CbE) and Acetylcholinesterase (AChE) of the hearts of *O. maya* adults.

PERMANOVA table of results

Source	df	SS	MS	Pseudo-F	P(perm)	Unique perms
Ac	2	5.7499	2.875	1.5109	0.259	997
Res	18	34.25	1.9028			
Total	20	40				

Details of the expected mean squares (EMS) for the model

Source	EMS
Ac	1*V(Res) + 6.381*S(Ac)
Res	1*V(Res)

Construction of Pseudo-F ratio(s) from mean squares

Source	Numerator	Denominator	Num.df	Den.df
Ac	1*Ac	1*Res	2	18

Estimates of components of variation

Source	Estimate	Sq.root
S(Ac)	0.15236	0.39033
V(Res)	1.9028	1.3794

Table S3. Description of the capture of adult female *Octopus maya* off the Sisal coast of the Yucatan Peninsula (21°9′55″N, 90°1′50″′W).

Group	Date of capture	Acclimation start date	Mitochondrial test date	Acclimation temperature	*n*
1	19-abr-21	26-abr-21	26-may-21	30°C	3
1	20-abr-21	27-abr-21	27-may-21	26°C	3
1	02-jun-21	09-jun-21	09-jul-21	24°C	4
2	18-oct-21	25-oct-21	25-nov-21	30° C	3
2	18-oct-21	25-oct-21	26-nov-21	30° C	3
2	22-oct-21	30-oct-21	30-nov-21	24°C	3
2	22-oct-21	30-oct-21	01-dic-21	24°C	3
3	01-may-22	07-may-22	07-jun-22	26°C	2
3	01-may-22	07-may-22	08-jun-22	26°C	3

Table S4. Biometric data of adult female *Octopus maya* used to assess mitochondrial function.

Capture month	ID	Total weight (g)	Systemic heart weight (g)	Total mitochondrial protein concentration (mg/ml)
jun-21	H 1_24°C	914.70	0.92	15.16
	H 2_24°C	964.50	0.95	15.13
	H 3_24°C	948.20	1.10	15.84
	H 4_24°C	1038.00	0.83	14.26
oct-21	H 7_24°C	1269.60	1.07	10.24
	H 8_24°C	1435.70	1.48	10.74
	H 9_24°C	1562.80	1.54	11.70
	H 10_24°C	1274.80	1.69	7.06
	H 11_24°C	802.50	0.82	9.83
	H 12_24°C	1489.80	1.30	10.55
abr-21	H 1_26°C	1006.90	1.36	13.16
	H 2_26°C	985.80	1.26	8.97
	H 3_26°C	1412.90	1.26	9.92
may-21	H 1_26°C	707.00	0.96	9.97
	H 2_26°C	525.00	0.68	10.05
	H 3_24°C	845.20	0.79	10.44
	H 4_24°C	918.00	1.02	9.32
	H 5_24°C	992.00	0.86	10.51
abr-21	H 1_30°C	782.50	0.78	14.60
	H 2_30°C	1200.00	1.55	14.97
	H 3_30°C	186.10	0.24	9.53
oct-21	H 1_30°C	1520.00	1.42	8.13
	H 2_30°C	1160.00	1.25	9.41
	H 3_30°C	1147.00	1.24	8.81
	H 4_30°C	1210.00	1.29	8.77
	H 5_30°C	1260.00	1.40	9.69
	H 6_30°C	1430.00	1.43	10.23

Biology Open • Supplementary information
